# Development and application of patient decision aids

**DOI:** 10.4178/epih/e2015018

**Published:** 2015-04-08

**Authors:** Jong-Myon Bae

**Affiliations:** Department of Preventive Medicine, Jeju National University School of Medicine, Jeju, Korea

**Keywords:** Decision aids, Thyroid neoplasms, Early detection of cancer, Clinical decision support systems

## Abstract

With the current overdiagnosis of thyroid cancer resulting from routine screening in Korea, it is necessary to educate the public that not all cancers are malignant. The exposure to patient decision aids (PtDAs) compared to usual care reduced the number of people choosing to undergo prostate-specific antigen screening. This article introduces the definition, usefulness, and developmental processes of PtDAs and suggests the urgent need for a Korean PtDA related to thyroid cancer screening.

## INTRODUCTION

An article published in a renowned international journal in 2014 [1] that raised concerns about overdiagnosis for thyroid cancer screening caused great waves of public anxiety at home and abroad. This study advanced a claim that ultrasonography screening is responsible for the rapid increase in the thyroid cancer incidence, placing it at the top of the list [2] without contributing to a reduction in mortality [3] and interpreted it as overdiagnosis through the unnecessary use of ultrasonography screening [1]. On a related note, an unfounded claim that the increase in thyroid cancer rates among women living near nuclear power plants is ascribable to the exposure to radiation [4] has been causing concerns across the country [5].

Debates over overdiagnosis in the screening process also surround prostate cancer, breast cancer, and skin melanoma [3,6]. One good example is the rapid increase in the incidence rate of prostate cancer in North America in early 2000 with the advent of prostate-specific antigen (PSA) testing in clinical screening settings [7]. The efforts made to solve the dilemma and overcome related controversies are significant as events that provide the opportunity for review and reflection [8]. Especially, Esserman & Thompson [8] emphasized the absolute necessity for vulnerable group education about screening adequacy. Despite the diagnostic principle that specific cancer screening testing is not recommended for the general population [9], this principle cannot be always respected in the face of the fear of those with wishing to be examined [10,11]. Since the ultimate goal of screening is the improvement in patient quality of life [12], the examinees’ values should be respected [11,13] in the shared decision making process [14-16].

PSA overdiagnosis-related debates and reflections resulted in the development of patient decision aids (PtDAs) were developed to help patients make adequate screening decisions [16]. According to a systematic review published in 2014, the application of these tools reduced the PSA test rate by 13% (summary relative risk, 0.87; 95% confidence interval, 0.77 to 0.98;

n=9) [17]. Based on this result, it may be assumed that if the development and application of a PtDA for the shared decision-making process for thyroid cancer screening would contribute to counteracting the overdiagnosis of thyroid cancer. Against this background, this paper reports on the development and application of a PtDA for cancer screening.

## BODY

### Definition of patient decision aids

The International Patient Decision Aid Standards (IPDAS) Collaboration, which initiated official activities in 2003 [18,19], defines PtDA as “tools designed to help people participate in decision making about health care options.” In other words, PtDA presents various options to patients in certain conditions to make well-informed final decisions from among possible options by weighing them according to their own personal value. From this active participation aspect, PtDAs are differentiated from educational materials to help patients better understand specific diseases or passive informed consent materials designed to obtain informed consent forms [17,20].

### Contents of patient decision aids

The contents of the PtDA developed by the American Society of Clinical Oncology [21,22] as tools for making decisions about the PSA test designed for the early detection of prostate cancer are divided in the following seven item clusters: (1) general introduction to the PSA test, (2) interpretation of the PSA test results, (3) possible options in the case of high PSA levels, (4) benefits and risks of the PSA testing, (5) personalized items for identifying individual values as decisional needs, (6) overall and final decision, and (7) reference materials. This exemplifies that a good PtDA presents a benefit-risk balance by providing disease information and allows systematic checking of the related elements that are likely to influence the final decision, concretizes individual value assessment levels, and gives examples of other cases with coaching throughout the stages of the decision-making process [13,16,17,20].

### Development of patient decision aids

All PtDAs developed worldwide to date are presented by countries in the clearinghouse system [14,20]. PtDAs on a variety of diseases have been developed in many different forms [14,23], and the current trendsetters are audiovisual materials and web-based programs for self-administrated tools that are designed to save time and medical personnel costs [13,20].

Table 1 outlines the purpose- and application-related usability of PtDAs extracted from the literature [13,17,19,20]. The main objectives of PDA development are to induce patients’ active participation in the decision-making process, enhance the understanding of the disease in question, reduce the stress related to decision making by facilitating consistent decision-making according to individual value assessments, and improve quality of decision-making process. On the other hand, they should also prevent patients from choosing multiple options and should not replace medical counseling or tools intended to enhance treatment compliance.

To accomplish these objectives, PtDAs should be developed in compliance with international standards [14,24,25] using meticulous planning and implementation processes. The IPDAS Collaboration presented a PtDA development process consisting of the following five steps (Table 2) [24]. (1) Clarification of the necessity for development: after clarifying the need to develop a PtDA, development objectives are set and information materials about clinical decisional options and their results necessary for decision-making are reviewed. (2) Constitution of the development committee (expert panel): the development committee is divided into focus groups participating in the actual development and steering group managing conflicts of interest. (3) Compilation: the PtDA is drafted after determining the presentation frames such as audiovisual materials or computational systems [26]. (4) Alpha testing and feedback process: the draft PtDA is applied to patients and their reactions are converged and reflected upon draft review. (5) Beta testing: opinions of external experts and patients are reflected in the final review. The developed PtDA is implemented [13] and updated to reflect newly acquired insights or decisional factors [14]. Due to this highly complicated and time-consuming process [14], PtDA development is implemented under ongoing international cooperation under the flag of the IPDAS Collaboration [27].

### Application of patient decision aids

A qualitative evaluation is essential for the efficient applications of existing PtDAs [14], so the IPDAS Collaboration presented a quality checklist [27], while the Canadian Ottawa Hospital Research Institute presented its own 19-item checklist [28]. On the other hand, there is a research need for investigation of the actual effects of the PtDAs whose validity has been established. According to the systematic review conducted by Stacey et al. [17], the impacts of the three papers reporting on the statistically significant effects of PtDA as a decision-making tool for PSA testing as prostate cancer screening were 9% to 42% (Table 3) [29-31]. This wide gap is indicative of the fact that the effects of PtDAs vary considerably due to differences in the health insurance systems among countries, efforts of health care personnel to introduce PtDAs, and acceptance among patients and caregivers.

## CONCLUSION AND SUGGESTIONS

Our main clearinghouse search as of January 2015 yielded no results regarding thyroid cancer screening PtDAs with validated efficacy [14,20,28]. Even in the presence of PtDAs developed in foreign countries, the development of PtDAs matching the conditions of the local population is indispensable. The main reason is that they should reflect the natural history and local epidemiological data of the diseases in question [22,28]. Moreover, the cost value perceived by the locals (willingness to pay) should be reflected [16,32] and the tool should be drafted in the individuals’ native language [33]. This justifies the compelling need for Korean health care researchers to develop a Korean PtDA to solve the present dilemma of thyroid cancer overdiagnosis.

PtDA development should ideally be promoted and implemented by a public institution such as the Korean National Evidence-based Health Care Collaborating Agency [32] rather than by a small group of researchers. Its application will require expansion by a clearinghouse operation. Additionally, affiliation to activities in the IPDAS Collaboration is necessary to accumulate PtDA development and evaluation experiences [18]. In the future, for other clinical conditions requiring decision-making support due to medical uncertainty [15,16,34,35] as well as thyroid cancer screening, the development of PtDAs tailored to the needs of Korean patients will reduce unnecessary medical resource expenditures [36,37] and inequity of inter-regional medical services [38]. Thus a good PtDA will contribute finally to quality enhancement of public health care [39].

## Figures and Tables

**Table 1.** Purposes of developing patient decision aids

	Description
Do	Improve patient decision quality
	Reduce decision conflict
	Increase participation in decision-making
	Help patient to make a choice that is consistent with their own values
	Improve people’s knowledge regarding options
	Reduce conflict related to feeling uninformed and unclear about personal values
Don’t	Advise patients to choose one option over another
	Intend to replace practitioner consultation
	Intend to increase treatment adherence

Modified from O'Connor A. ACP J Club 2001;135:A11-A12 [13]; Stacey D, et al. Cochrane Database Syst Rev 2014;1:CD001431 [17]; Volk RJ, et al. BMC Med Inform Decis Mak 2013;13 Suppl 2:S1 [19]; Ng CJ, et al. Australas Med J 2013;6:95-99 [20].

**Table 2.** Five steps of developing patient decision aids

Step	Main activity
1	Scope problems
2	Organize committee
3	Develop draft
4	Test usability
5	Test feasibility

Modified from Coulter A, et al. BMC Med Inform Decis Mak 2013;13 Suppl 2:S2 [24].

**Table 3.** Effect sizes of decision aids for prostate cancer screening

Author [reference]	Decision aid type	Difference size
Frosch et al. [29]	Traditional	9.1 (%)
Volk et al. [30]	Educational videotape	10.9 (%)
Wolf et al. [31]	Scripted informational intervention	0.8 (on five-point scale)

Three results showing significant reductions in screening among selected articles in the systematic review conducted by Stacey D, et al. Cochrane Database Syst Rev 2014;1:CD001431 [17].
