# Supplementary material for: Development and application of patient decision aids
Source: Epidemiol Health. 2015 Apr 8;37:e2015018. doi: 10.4178/epih/e2015018 (PMC4430759; doi:10.4178/epih/e2015018)
Supplement: Supplementary file 1 [file epih-37-e2015018-supplementary.pdf]

<연구방법>

환자결정 조력도구: 개발 및 적용

Development and Application of Patients Decision Aids

Abstract

While suggesting overdiagnosis for thyroid cancer screening in Korea lately, it should be necessary to educate people to understand that not all cancers have the potential to kill. It is hopeful to solve the overdiagnosis dilemma that exposure to patient decision aids (PtDA) compared to usual care reduced the number of people choosing to have prostate-specific antigen screening. This article introduced definition, usefulness and developmental processes of the PtDA and suggested urgently on developing a PtDA related to thyroid cancer screening being fitted for Koreans.

Key words: Decision aids, thyroid neoplasms, early detection of cancer, Clinical decision support systems

1. 서론

2014년도에 갑상선암의 조기검진에 있어 과잉진료의 우려를 제기한 논문이 저명한 국제 학술지에 게재되면서[1], 국내외적으로 큰 파장을 일으켰다. 주된 주장은 해당 암의 발생률이 급격히 증가하여 발생순위 1위를 차지한 가운데[2] 사망률 감소에는 기여하지 않았다는 점[3]에서 불필요한 초음파 진단에 의한 과진단 (over-diagnosis)이 이루어 졌다고 해석하고 있다[1]. 한편에서는 원자력발전소 주변주민 중 여성의 갑상선암 발생증가[4]가 방사선노출에 의한 것이라는 근거 부족의 일방적 주장은 일반 국민들을 불안하게 만들고 있다[5].

암의 조기검진 과정에서 과잉진단에 대한 논란은 갑상선암 뿐만 아니라 전립선암, 유방암, 피부흑색종 등에서도 있었다[3,6]. 특히 2000년 초 혈중 전립선특이항원 (prostate specific antigen, 이하 PSA)이 검진현장에 도입되면서 전립선암의 발생률이 급증했던 북미의 경험들[7]과 모순점들을 극복하려는 노력[8]을 살펴보는 것은 의미가 크다. 이중 Esserman & Thompson [8]은 검진 대상자에게 검진의 적절성에 대한 교육을 절대적으로 강조하였다. 실제로 일반인에 있어 특정 암 검진은 권장되지 않는다는 진료지침이 있음에도 불구하고[9], 검진자가 해당 암에 대한 공포를 극복하지 못한다면 근거를 무시하고 시행할 수밖에 없기 때문이다[10,11]. 검진의 궁극적인 목표는 당사자의 삶의 질 (Quality of Life)을 향상시키는 것이기에[12], 검진과정에 있어서 검진자의 가치를 반영한 가운데[11,13], 참여에 의한 의사결정 (Shared Decision Making)이 필요한 것이다[14,15,16].

이에 따라, PSA 수치에 따라 검진자의 결정을 어떻게 할 것인가를 도와주는 환자결정조력도구 (Patient Decision Aids, 이하 PtDA)들이 개발되었고[16], 이들 도구들을 활용했을

때 PSA 검진율을 13% (summary RR = 0.87; 95% CI 0.77 to 0.98; n = 9) 줄인다는 체계적 고찰 연구가 2014년도에 발표되었다[17]. 이 결과를 근거할 때, 갑상선암 검진에 대한 PtDA를 개발하여 적용한다면 해당 암의 과잉진단을 막을 수 있다고 예상할 수 있겠다. 따라서 본 원고는 PtDA의 개발과 활용에 대하여 살펴보고자 한다.

2. 본론

가. 환자결정조력도구 정의

2003년부터 공식 활동을 시작한 The International patient Decision Aid Standards Collaboration (이하 IPDAS 연합)[18,19]은 PtDA를 보건의료 상황에서 당사자가 의사결정에 참여하도록 도와주는 도구 (tools designed to help people participate in decision making about health care options.) 라고 정의하고 있다. *이 정의를 부연설명하자면, PtDA는 특정한 의료상황에 부딪쳤을 때 환자들이 선택할 수 있는 여러 가지 방안(options)들을 담아 제시하여, 환자 각자가 개별적인 가치 판단 (the personal value)에 따라 검토하고 최종 의사결정을 할 수 있도록 해주는 도구이다.* 이 점에서, 특정 질병의 이해를 돕는 각종 환자교육 자료 (educational materials)나 동의서를 받기 위한 설명 자료 (passive informed consent materials)와는 차별된다[17,20].

나. 환자결정조력도구 항목

전립선암 조기검진을 위해 시행하는 전립선특이항원 (Prostate-specific antigen, PSA) 검사 시행여부를 결정하기 위하여 미국임상암학회 (American Society of Clinical Oncology, ASCO)가 개발한 PtDA [21,22]에 담긴 내용을 살펴보면, (1) PSA 검사에 대한 전반적인 소개 (2) PSA 검사 결과의 해석 내용 (3) PSA 수치가 높게 나왔을 때 취할 수 있는 경우들 (4) PSA 검사의 이득 (benefits)과 유해 (risks) (5) 개인별 판단가치를 정할 수 있는 개별 질문 문항 제시 (6) 종합 및 최종 결정 (7) 참고할 문헌들 소개 항목으로 이루어져있다. 따라서 잘 개발된 PtDA는 질병에 대한 정보를 바탕으로 선택에 따른 득실을 제시하면서, 최종 결

과에 미칠 관련 요소들을 개별적으로 점검토록 하고, 각자의 가치판단 수준을 구체화 시키고, 다른 사례의 예를 들면서 선택 과정 단계마다 조언 (coaching)을 하는 내용을 담는다 [13,16,17,20].

다. 환자결정조력도구 개발

지금껏 전세계적으로 개발된 PtDA는 나라별로 clearinghouse 체제로 정리되어 제시하고 있다[14,20]. 그동안 여러 질병에 있어 다양한 형태로 개발되어 왔으며[14,23], 최근에는 의료진의 인력과 시간을 절약한다는 점에서 자기 응답식 (self-administrated) 진행을 할 수 있도록 시청각 자료나 컴퓨터 웹 프로그램이 대세이다[13,20].

기존 문헌들에서 PtDA의 개발 목적과 적용에 따른 유용성에 관한 내용들은 Table 1로 정리하였다[13,17,19,20]. *주된 개발 목적은 환자의 의사결정 과정에 있어 능동적인 참여를 유도하며, 해당 질병에 대한 이해를 높이고, 개인의 가치판단에 따라 일관된 결정을 할 수 있게 하여 결정의 갈등을 줄이고 결정과정의 질적 수준을 향상시킬 수 있도록 해야 한다. 반면 여러 개의 선택을 하지 않도록 해야 하고, 의사의 자문을 대신하거나 치료의 순응도를 높이기 위한 도구로 사용해서는 안된다.*

이러한 목적들을 달성하려면 국제적인 제시 기준[14,24,25]에 맞추어 치밀한 기획과 과정을 밟아서 개발해야 한다. IPDAS 연합이 제시한 개발과정은 5 단계로 제시하고 있다 [24] (Table 2). 1단계는 개발 필요성 확인단계로써, 개발의 필요성을 확인하고 개발 목적을 결정한 다음, 임상 결정의 선택 옵션들과 각각의 결과들, 선택에 필요한 정보들을 알아본다. 2단계는 개발 위원회를 구성하는 단계로, 실제 개발에 참여하는 focus groups과 이해상충을

조율하는 steering group으로 나눈다. 3단계는 시청각 자료 혹은 전산 시스템 등 [26]과 같이 제시할 틀을 결정한 다음, 이에 맞추어 초안 (draft)을 만드는 과정이다. 4단계는 환자에게 실제 적용해서 (alpha testing) 그 반응을 수용하고 이를 반영하여 내용을 다듬는 과정이다. 마지막 5단계는 개발에 참여하지 않는 외부 전문가들이나 일반인의 의견을 반영하는 (beta testing) 마무리 단계이다. 이후 개발된 것을 확산하는 과정 (implementing) [13]과, 새로운 근거들을 반영하는 개정과정 (updating)이 추가로 요구된다[14]. 이상의 개발과정은 매우 복잡하고 장시간을 요구하기 때문에[14], IPDAS 연합을 중심으로 국제적인 공조로 진행하고 있다[27].

라. 환자결정조력도구 적용

기존에 개발된 PtDA를 적용하기 위해서는 질적 평가 (evaluation)가 필요한데[14], IPDAS가 제시한 것[27]뿐만 아니라, 캐나다 Ottawa Hospital Research Institute 는 19가지 점검 항목을 제시하고 있다[28].

한편 타당성을 확보한 PtDA를 적용시 그 효과가 어느 정도인가에 대하여도 많은 연구가 필요하다. Stacey et al. [17] 이 수행한 체계적 고찰 내용 중, 전립선암 조기검진용 PSA 검사 시행 결정을 도와주는 PtDA 도구가 통계적으로 유의한 효과가 있다고 보고한 3가지 논문의 효과 크기는 9%에서 42% 이었다[29-31] (Table 3). 이렇게 큰 범위를 갖는 것은 PtDA 도구를 적용하는 해당 국가의 보건의료 시스템의 차이, 보건의료진의 현장도입 노력의 차이, 환자 및 보호자의 수용 정도 차이 등에 따라서 그 효과가 다를 수 있기 때문이다.

3. 결론 및 제언

2015년 1월 현재 주요 clearninghouse를 방문하여 검색을 하였을 때, 갑상선암 검진에 관한 것으로 효과성까지 입증된 PtDA는 없었다[14,20,28]. 설사 외국에서 개발된 것이 있다 하더라도, 자국민에 맞는 PtDA 개발이 요구될 수 밖에는 없다. 그 이유는 내용상 특정 질병의 자연사와 역학통계 정보를 담아야 하며[22,28], 선택에 있어 그 나라 국민의 비용가치 –비용지불의사 (willing to pay) 등-를 반영해야 하며[16,32], 자국민 모국어로 된 도구여야만 하기 때문이다[33]. 결국 한국의 갑상선암의 과잉진단의 문제 해결을 위하여 한국의 보건의료 연구자가 앞장서서 PtDA 개발을 해야만 한다.

그러나 PtDA 개발에 있어 몇몇 연구자가 주축이 되기 보다는, 한국보건의료연구원[32] 같이 국가연구기관이 주축이 되어 개발을 추진하고 clearinghouse를 운영하여 확산하는 것이 바람직할 것이다. 그리고 PtDA 개발 및 평가 경험을 축적하기 위하여 IPDAS 연합에 가입하여 활동하는 것도 필요하다[18]. 향후 갑상선암 조기검진 뿐만 아니라, 의료의 불확실성 때문에 선택결정이 필요한 임상상황에 있어[15,16,34,35] 우리나라 환자에 맞는 PtDA가 개발된다면, 불필요한 의료자원 낭비를 줄일 수 있고[36,37], 지역간 의료제공의 형평성이 확보되는 등[38] 보건의료의 질적 향상에도 직접 기여하게 될 것이다[39].

감사의 글

이 논문은 2014학년도 제주대학교 학술진흥연구비 지원사업에 의하여 연구되었음

References

1. Ahn HS, Kim HJ, Welch HG. Korea's thyroid-cancer "epidemic"--screening and overdiagnosis. N Engl J Med 2014;371:1765-1767.

2. Jung KW, Won YJ, Kong HJ, Oh CM, Lee DH, Lee JS. Cancer statistics in Korea: incidence, mortality, survival, and prevalence in 2011. Cancer Res Treat 2014;46:109-123.

3. Bae JM. Overdiagnosis: Concepts and Estimation. Epidemiol Health 2015;37:e2015004.

4. Ahn YO, Li ZM; KREEC Study Group. Cancer risk in adult residents near nuclear power plants in Korea - a cohort study of 1992-2010. J Korean Med Sci 2012;27:999-1008.

5. Bae JM. A case report about doubting a misconduction of publication by a third party based on the confusing ownership of databases. J Med Life Sci 2014;11:77-81. (Korean)

6. Welch HG, Black WC. Overdiagnosis in cancer. J Natl Cancer Inst 2010;102:605-613.

7. Etzioni R, Penson DF, Legler JM, di Tommaso D, Boer R, Gann PH, et al. Overdiagnosis due to prostate-specific antigen screening: lessons from U.S. prostate cancer incidence trends. J Natl Cancer Inst 2002;94:981-990.

8. Esserman L, Thompson I. Solving the overdiagnosis dilemma. J Natl Cancer Inst 2010;102:582-583.

9. Suh M, Choi KS, Lee YY, Jun JK. Trends in Cancer Screening Rates among Korean Men and Women: Results from the Korean National Cancer Screening Survey, 2004-2012. Cancer Res Treat 2013;45:86-94.

10. Kramer KM, Bennett CL, Pickard AS, Lyons EA, Wolf MS, McKoy JM, et al. Patient preferences in prostate cancer: a clinician's guide to understanding health utilities. Clin Prostate Cancer 2005;4:15-23.

11. Lee DW, Neumann PJ, Rizzo JA. Understanding the medical and nonmedical value of diagnostic testing. Value Health 2010;13:310-314.

12. Gomella LG, Johannes J, Trabulsi EJ. Current prostate cancer treatments: effect on quality of life. Urology 2009;73(5 Suppl):S28-S35.

13. O'Connor A. Using patient decision aids to promote evidence-based decision making. ACP J Club

2001;135:A11-A12.

14. Lenz M, Buhse S, Kasper J, Kupfer R, Richter T, Mühlhauser I. Decision aids for patients. Dtsch Arztebl Int 2012;109:401-408.

15. Bae JM. The clinical decision analysis using decision tree. Epidemiol Health 2014;36:e2014025.

16. Bae JM. Value-based medicine: concepts and application. Epidemiol Health 2015;37:e2015014.

17. Stacey D, Légaré F, Col NF, Bennett CL, Barry MJ, Eden KB, et al. Decision aids for people facing health treatment or screening decisions. Cochrane Database Syst Rev 2014;1:CD001431.

18. International Patient Decision Aid Staandards Collaboration. What are patient decision aids? [cited 2014 Jan 8]. Available from: http://ipdas.ohri.ca/what.html.

19. Volk RJ, Llewellyn-Thomas H, Stacey D, Elwyn G. Ten years of the International Patient Decision Aid Standards Collaboration: evolution of the core dimensions for assessing the quality of patient decision aids. BMC Med Inform Decis Mak 2013;13 Suppl 2:S1.

20. Ng CJ, Lee YK, Lee PY, Abdullah KL. Health innovations in patient decision support: Bridging the gaps and challenges. Australas Med J 2013;6:95-99.

21. American Society of Clinical Oncology. Screening for prostate cancer with prostate-specific antigen (PSA) testing. [cited 2014 Jan 8]. Available from: http://www.instituteforquality.org/screening-prostate-cancer-prostate-specific-antigen-psa-testing-american-society-clinical-oncology

22. Basch E, Oliver TK, Vickers A, Thompson I, Kantoff P, Parnes H, et al. Screening for prostate cancer with prostate-specific antigen testing: American Society of Clinical Oncology Provisional Clinical Opinion. J Clin Oncol 2012;30:3020-3025.

23. Leatherman S, Warrick L. Effectiveness of decision aids: a review of the evidence. Med Care Res Rev 2008;65(6 Suppl):79S-116S.

24. Coulter A, Stilwell D, Kryworuchko J, Mullen PD, Ng CJ, van der Weijden T. A systematic development process for patient decision aids. BMC Med Inform Decis Mak 2013;13 Suppl 2:S2.

25. Elwyn G, O'Connor AM, Bennett C, Newcombe RG, Politi M, Durand MA, et al. Assessing the quality of

decision support technologies using the International Patient Decision Aid Standards instrument (IPDASi). PLoS One. 2009;4:e4705.

26. Hirsch O, Keller H, Krones T, Donner-Banzhoff N. Acceptance of shared decision making with reference to an electronic library of decision aids (arriba-lib) and its association to decision making in patients: an evaluation study. Implement Sci 2011;6:70.

27. Elwyn G, O'Connor A, Stacey D, Volk R, Edwards A, Coulter A, et al. Developing a quality criteria framework for patient decision aids: online international Delphi consensus process. BMJ 2006;333:417.

28. Ottawa hosptial Research Institute. Patient decision aids. [cited 2014 Jan 8]. Available from: http://decisionaid.ohri.ca/AZsearch.php?

29. Frosch DL, Bhatnagar V, Tally S, Hamori CJ, Kaplan RM. Internet patient decision support: a randomized controlled trial comparing alternative approaches for men considering prostate cancer screening. Arch Intern Med 2008;168:363-369.

30. Volk RJ, Spann SJ, Cass AR, Hawley ST. Patient education for informed decision making about prostate cancer screening: a randomized controlled trial with 1-year follow-up. Ann Fam Med 2003;1:22-28.

31. Wolf AM, Nasser JF, Wolf AM, Schorling JB. The impact of informed consent on patient interest in prostate-specific antigen screening. Arch Intern Med 1996;156:1333-1336.

32. Ahn JH, Kim YH, Shin SJ, Park JY. The Asian Collaborative Study of cost-effectiveness in healthcare decsion making; 2012 [cited 2014 Jan 8]. Available from: http://www.neca.re.kr/center/researcher/report_view.jsp?boardNo=GA&seq=65&q=626f6172644e6f3d4741.

33. Charles C, Gafni A, Whelan T, O'Brien MA. Treatment decision aids: conceptual issues and future directions. Health Expect 2005;8:114-125.

34. Bae JM. Academic strategies based on evidence-practice gap. Hanyang Med Rev 2015;35:3-8. (korean)

35. Stacey D, Samant R, Bennett C. Decision making in oncology: a review of patient decision aids to support patient participation. CA Cancer J Clin 2008;58:293-304.

36. O'Connor AM, Llewellyn-Thomas HA, Flood AB. Modifying unwarranted variations in health care: shared decision making using patient decision aids. Health Aff (Millwood) 2004;Suppl Variation:VAR63-VAR72.

37. Döring AC, Hageman MG, Mulder FJ, Guitton TG, Ring D; Science of Variation Group; et al. Trigger finger: assessment of surgeon and patient preferences and priorities for decision making. J Hand Surg Am 2014;39:2208-2213.

38. Reames BN, Shubeck SP, Birkmeyer JD. Strategies for reducing regional variation in the use of surgery: a systematic review. Ann Surg 2014;259:616-627.

39. O'Connor AM, Wennberg JE, Legare F, Llewellyn-Thomas HA, Moulton BW, Sepucha KR, et al. Toward the 'tipping point': decision aids and informed patient choice. Health Aff (Millwood) 2007;26:716-725.

Table 1. The purposes of developing patient decision aids*

	descriptions
Do	
	improve patient decision quality
	reduce decision conflict
	increase participation in decision making
	help patient to make a choice that is consistent with their own values
	improve people's knowledge regarding options
	reduce decisional conflict related to feeling uninformed and unclear about their personal values
Do Not	
	advise to choose one option over another
	mean to replace practitioner consultation
	increase treatment adherence

* modified from Reference number [13, 17, 19, 20]

Table 2. Five steps of developing patient decision aids*

Step	Main activities
1	Scoping Problems
2	Organizing Committee
3	Developing Draft
4	Testing Usability
5	Testing Feasibility

* modified from Reference number [24]

Table 3. Effect sizes of decision aids for prostate cancer screening *

Author [Reference	Kinds of decision aids	Difference/Change size
Frosch [29]	Traditional decision aid	9.1(%)
Volk [30]	Educational videotape	10.9(%)
Wolf [31]	Scripted Informational intervention	0.8 (on 5-point scale)

* Three results showing significant reduction in screening among selected articles on the systematic review conducted by Stacey et al. [17]
